# Critical Orientation in the Jungle of Currently Available Methods and Types of Data for Source Attribution of Foodborne Diseases

**DOI:** 10.3389/fmicb.2019.02578

**Published:** 2019-11-12

**Authors:** Lapo Mughini-Gras, Pauline Kooh, Philippe Fravalo, Jean-Christophe Augustin, Laurent Guillier, Julie David, Anne Thébault, Frederic Carlin, Alexandre Leclercq, Nathalie Jourdan-Da-Silva, Nicole Pavio, Isabelle Villena, Moez Sanaa, Laurence Watier

**Affiliations:** ^1^Centre for Infectious Disease Control, National Institute for Public Health and the Environment, Bilthoven, Netherlands; ^2^Faculty of Veterinary Medicine, Institute for Risk Assessment Sciences, Utrecht University, Utrecht, Netherlands; ^3^Department of Risk Assessment, French Agency for Food, Environmental and Occupational Health and Safety, Maisons-Alfort, France; ^4^Research Chair in Meat-Safety, Faculty of Veterinary Medicine, University of Montreal, Saint-Hyacinthe, QC, Canada; ^5^Ecole Nationale Vétérinaire d’Alfort, Maisons-Alfort, France; ^6^Laboratory for Food Safety, French Agency for Food, Environmental and Occupational Health and Safety, Maisons-Alfort, France; ^7^Ploufragan-Plouzané Laboratory, French Agency for Food, Environmental and Occupational Health and Safety, Ploufragan, France; ^8^UMR 408 SQPOV “Sécurité et Qualité des Produits d’Origine Végétale” INRA, Avignon Université, Avignon, France; ^9^Institut Pasteur, Biology of Infection Unit, National Reference Centre and WHO Collaborating Centre for Listeria, Paris, France; ^10^Santé Publique France (French National Public Health Agency), Saint-Maurice, France; ^11^Laboratory for Animal Health, French Agency for Food, Environmental and Occupational Health and Safety, Maisons-Alfort, France; ^12^Laboratory of Parasitology-Mycology, EA ESCAPE, University of Reims Champagne-Ardenne, Reims, France; ^13^Department of Biostatistics, Biomathematics, Pharmacoepidemiology and Infectious Diseases (B2PHI), Institut National de la Santé et de la Recherche Médicale (INSERM), UVSQ, Institut Pasteur, Université Paris-Saclay, Paris, France

**Keywords:** source attribution, foodborne pathogen, epidemiological studies, typing methods, frequency-matching models, population genetics models, quantitative risk assessment, expert knowledge elicitation

## Abstract

With increased interest in source attribution of foodborne pathogens, there is a need to sort and assess the applicability of currently available methods. Herewith we reviewed the most frequently applied methods for source attribution of foodborne diseases, discussing their main strengths and weaknesses to be considered when choosing the most appropriate methods based on the type, quality, and quantity of data available, the research questions to be addressed, and the (epidemiological and microbiological) characteristics of the pathogens in question. A variety of source attribution approaches have been applied in recent years. These methods can be defined as top–down, bottom–up, or combined. Top–down approaches assign the human cases back to their sources of infection based on epidemiological (e.g., outbreak data analysis, case-control/cohort studies, etc.), microbiological (i.e., microbial subtyping), or combined (e.g., the so-called ‘source-assigned case-control study’ design) methods. Methods based on microbial subtyping are further differentiable according to the modeling framework adopted as frequency-matching (e.g., the Dutch and Danish models) or population genetics (e.g., Asymmetric Island Models and STRUCTURE) models, relying on the modeling of either phenotyping or genotyping data of pathogen strains from human cases and putative sources. Conversely, bottom–up approaches like comparative exposure assessment start from the level of contamination (prevalence and concentration) of a given pathogen in each source, and then go upwards in the transmission chain incorporating factors related to human exposure to these sources and dose-response relationships. Other approaches are intervention studies, including ‘natural experiments,’ and expert elicitations. A number of methodological challenges concerning all these approaches are discussed. In absence of an universally agreed upon ‘gold’ standard, i.e., a single method that satisfies all situations and needs for all pathogens, combining different approaches or applying them in a comparative fashion seems to be a promising way forward.

## Introduction

Source attribution of foodborne diseases is defined as the partitioning of the human cases caused by foodborne pathogens among their animal, food, and environmental reservoirs and/or transmission routes (Pires et al., 2009). Estimating the relative contributions of different sources to the human disease burden is crucial to set priorities for food safety interventions and to measure the impact of such interventions.

Source attribution has become an ‘umbrella term’ that includes a growing number of methodological approaches and types of data (Pires et al., 2009). A detailed overview of definitions and terminology for source attribution is available elsewhere (Pires et al., 2009; Wagenaar et al., 2013). In brief, for pathogens of animal origin, the animals are usually defined as reservoirs (i.e., amplifying hosts in which the pathogen normally lives and multiplies). Food, the environment, direct contact with animals, etc. are examples of transmission routes, whereas meat, eggs, milk, etc. are examples of exposures, and consumption of raw meat, swimming in open water, living in a farm, etc. are examples of risk factors (Wagenaar et al., 2015). In practice, however, the term ‘source’ refers to any of these points across the transmission chain.

A variety of source attribution methods has been developed for foodborne pathogens. Conceptually, these methods can be defined as ‘top–down,’ ‘bottom–up,’ or combined. Top–down approaches assign the human cases (i.e., the ‘top’ of the transmission chain) back to their sources of infection (i.e., the ‘bottom’). They correspond to: (1) epidemiological methods, e.g., analysis of outbreak investigations (Pires et al., 2010; Batz et al., 2012; Painter et al., 2013) and case-control/cohort studies of sporadic infections (Domingues et al., 2012; Kintz et al., 2017), (2) microbiological methods, i.e., based on microbial subtyping (Barco et al., 2013), or (3) combination of both (Mughini Gras et al., 2012; Mughini-Gras et al., 2014b, 2018b; Mossong et al., 2016; Rosner et al., 2017). In addition, source attribution based on microbial subtyping can be further differentiated according to the computational modeling framework adopted: (1) frequency-matching models (Hald et al., 2004; Mullner et al., 2009a; Pires and Hald, 2010; David et al., 2013a; Mughini-Gras et al., 2014b, c), and (2) population genetics models (Pritchard et al., 2000; Wilson et al., 2008; Sheppard et al., 2009; Strachan et al., 2009; Mughini Gras et al., 2012, Mughini-Gras et al., 2014c; Miller et al., 2017). Conversely, bottom–up approaches like comparative exposure assessment (Pintar et al., 2016) predict the number of human cases caused by each source starting from the bottom, i.e., the level of contamination (prevalence and concentration) with a given pathogen in a source, and then go upwards in the transmission chain incorporating factors like human exposure to these sources and dose-response relationships. Other source attribution approaches are intervention studies, sometimes also referred to as ‘natural experiments’ (Vellinga and Van Loock, 2002; van Pelt et al., 2004; Sears et al., 2011; Tustin et al., 2011; Friesema et al., 2012) and expert knowledge elicitation (Havelaar et al., 2008; Butler et al., 2015a; Hald et al., 2016).

**FIGURE 1:**
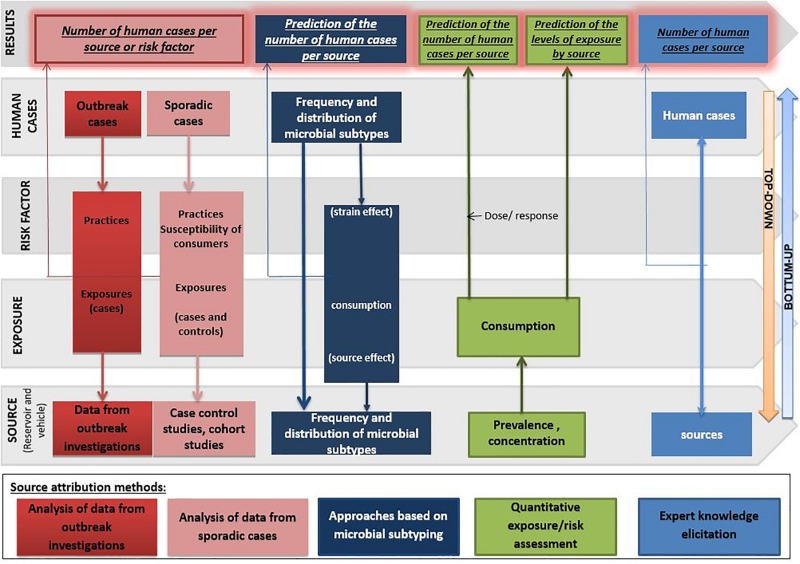
General overview of the different source attribution approaches for foodborne diseases.

Each approach has advantages and disadvantages to be considered when performing and/or interpreting a source attribution analysis (Figure 1). Herewith, we critically reviewed the currently available methods and data types for source attribution of foodborne pathogens, discussing their main strengths and weaknesses in order to guide readers toward the most appropriate methods based on the type, quality and availability of data, the food safety questions to be addressed, and the (epidemiological and microbiological) characteristics of the pathogens in question.

## Epidemiological Approaches

### Epidemiological Studies of Sporadic Foodborne Infections

Human cases of a foodborne disease may occur sporadically, or clustered as part of an outbreak. Case-control and cohort studies are used extensively to identify risk factors for infection. The principle is to compare the frequency of exposure to a given (risk) factor in a group of cases to the frequency of exposure in a control group (case-control study design), or to compare the frequency of case occurrence among those exposed to a given risk factor *vs*. an unexposed group (cohort study design).

Case-control studies are used more often for attribution of sporadic foodborne infections. Case-control studies are subject to several types of bias (e.g., selection bias, classification bias, recall bias, etc.), possibly differential for cases and controls. Ideally, the group of patients should be representative of all cases occurring in the population, and the control group should be at risk of acquiring the disease upon exposure, i.e., originate from a population with the same characteristics as the one where the patients come from. Deviation from this basic principle results in selection bias. In some studies, cases and controls are matched by age, gender, place of residence, etc. to control for potential confounding effects and gain analytical efficiency. Selection of several controls per case allows for increased statistical power, but the associated study costs rise in parallel (Fullerton et al., 2012).

Case definition is usually based on clinical symptoms and/or laboratory testing results, which may also include pathogen characterization. Collecting data on exposure to putative risk factors is usually retrospective in nature and mainly performed using questionnaires or interviews, with the recall period usually being the maximum duration of the incubation period for the disease in question. The investigated risk factors are often those that may have caused or favored transmission of the pathogen; that is, consumption of specific food items and general eating habits, underlying (possibly chronic) health conditions, behavioral or seasonal factors, etc. Typically, questionnaires are built on the findings of previous studies, possibly including new hypotheses (exposures) to be tested. These are comparatively more generic than the food items listed in the so-called ‘trawling questionnaires’ used in outbreak investigations, which are constructed to rapidly target specific food products to withdraw from the market.

The usual measure of association between the disease and the risk factors is the Odds Ratio (OR), whose interpretation for source attribution is not as direct as the Relative Risk (RR), although there are methods to convert them to one another (Grant, 2014). Logistic regression modeling is often the statistical method of choice for analyzing case-control study data. When a biologically plausible causal link between exposure and disease exists, calculating a Population Attributable Fraction (PAF) can provide an estimate of the number of cases attributable to a given risk factor (i.e., source) in question; that is, the cases that may be averted by eliminating exposure to such a risk factor (Stafford et al., 2008). Systematic reviews and meta-analyses of published case-control studies are useful to provide pooled ORs to identify the main risk factors for foodborne infections (Domingues et al., 2012; Kintz et al., 2017). They may also help increasing the number of sources to be considered and overcome the geographical and temporal limitations inherent of individual case-control studies.

In summary, case-control studies of sporadic foodborne diseases can provide valuable insights into determinants and risk factors for infection at the point of exposure (Hedberg, 2012; Fullerton and Mahon, 2013). Although these studies are challenged by several biases, recent examples show their usefulness in pinpointing specific sources. For instance, the link between consumption of ‘cantaloupe’ melons and *L. monocytogenes* infection in the United States of America (USA) was first suggested by a case-control study of sporadic cases (Varma et al., 2007). These studies are particularly useful for attribution of diseases that are sporadic in nature, such as campylobacteriosis (Kapperud et al., 2003; Wagenaar et al., 2015).

### Outbreak Investigations

Rapid collection of information about the possible causes of a foodborne outbreak, soon after case identification, favors the implementation of measures to prevent and finally stop the spread of the disease. Indeed, a foodborne outbreak investigation aims at identifying the contaminated food items, so that appropriate measures (e.g., recall of retailed products) can be implemented and lessons for prevention and management of future outbreaks can be drawn. During a typical foodborne outbreak investigation, health authorities would essentially collect three types of data: (1) epidemiologic data (e.g., spatiotemporal and sociodemographic distribution of cases, foods consumed and other exposures); (2) trace-back data (e.g., common points of contamination in the distribution chain of suspected foods, findings from food safety inspections in production facilities); (3) microbiological data (e.g., laboratory detection and possible subtyping of the pathogens in question from human cases, food, animal and/or environmental samples). The analysis of epidemiological data may include testing hypotheses in a case-control study or a retrospective cohort study, which allows for a comparison of the frequency of occurrence of the disease among consumers and non-consumers of suspected foods. Retrospective cohort studies are often used in outbreaks affecting confined communities, such as schools, hospitals, nursing homes, mass-gathering events, etc. Recently, there have been methodological developments in outbreak investigation settings, particularly in the USA, that move away from community-based case-control studies, such as the increasing use of case-cluster interviews to compare case-patients reporting consumption of specific foods before illness onset to population expected values (Laughlin et al., 2019). Microbiological data may provide strong evidence for the identification of the source of a foodborne outbreak, as infection with specific pathogen subtypes may be a criterion for definition and exclusion of cases that are not infected with the outbreak clone. For the purpose of source attribution, foodborne outbreaks (and individual cases therein) caused by a given pathogen in a defined time period are usually categorized by transmission route (e.g., food, direct contact with animals, person-to-person, etc.), and then further categorized by food source (e.g., chicken meat, eggs, dairy, etc.). The use of a standardized food classification system is strongly advocated (Painter et al., 2009). The broadness of defined food groups depends on the original resolution of available data and the objectives of the attribution assessment. Some methods allow considering the different ingredients of composite meals, which outbreaks can then be attributed to (Painter et al., 2009; Pires et al., 2010, 2012). The results are usually presented as the proportion of outbreaks (or outbreak cases) attributable to each source. This proportion can provide an estimate of the relative contributions of different food sources to the human disease burden. However, it should be kept in mind that outbreaks usually provide a partial picture of all infections occurring in the population (Pires et al., 2009).

In summary, outbreak data may cover a wide range of food sources and pathogens, so their analysis has the potential to provide insights for source attribution. However, data completeness is dependent on the quality and coverage of the surveillance system. Outbreaks of severe diseases, those caused by rare pathogens, and those with a high attack rate have a higher chance of being identified, thoroughly investigated and reported, whereas outbreaks of mild diseases, involving a few people and/or caused by less virulent pathogens, are more likely to remain unascertained and uninvestigated.

In absence of an investigation, the identification of the source of the outbreak relies on anecdotal information or on information supplied by, e.g., physicians, patients themselves, etc. In these cases, the strength of the evidence linking an outbreak to a given source is weak and tends to confirm notoriously high-risk foods, with possible overestimation of their contributions. The burden of foodborne pathogens that are sporadic in nature (e.g., *Campylobacter*, *Toxoplasma*, etc.) is poorly estimated by source attribution based on outbreak data, and other approaches (e.g., case-control studies) are preferable.

## Approaches Based on Microbial Subtyping

### Brief Overview of Typing Methods Applied to Foodborne Pathogens

Typing methods for foodborne pathogens have been developed with the main objective to characterize and compare strains with one another, as to determine their distinctive features, origins and similarities. The two main families of typing methods for microorganisms are based on either their expressed phenotype or on the nucleotide composition (genotype) of their full or partial genome. For *Salmonella*, two reviews encompass general considerations described in this section (Barco et al., 2013; Ferrari et al., 2017).

#### Phenotyping Methods

Biotyping is the basis of speciation using traditional microbiological tests for bacteria already defined at the genus, species, and subspecies levels. This involves identifying metabolic pathways (e.g., sugar utilization), specific enzyme activities (e.g., lysine decarboxylation, etc.) or biological, physiological or biochemical properties (e.g., Gram staining, mobility, etc.) of a given bacterium. When at least one or several characteristics are specific for a group within a species/subspecies (e.g., *Listeria ivanovii* subsp. *ivanovii*), then a specific biotype (e.g., *Shigella sonnei* biotype G ornithine decarboxylase positive) can be determined.

Serotyping describes the antigenic diversity within microorganisms. By means of agglutination using hyper-immune sera, the aim is to detect which antigens among those determining the serotype pattern are present on the tested strains. The sera used are directed against variable cell-wall antigens within the species of bacteria that possess them (poly-O chains of LPS for Gram-negative bacteria, polysaccharides or capsules for Gram-negative and Gram-positive bacteria, called factor O, and against the flagella antigens, called factor H, and rarely capsular antigens on the surface of some enteric bacilli, called factor K). An updated scheme that identifies the antigenic diversity present in the species, and the way to characterize a specific serotype, is a prerequisite, e.g., the White-Kauffmann-Le Minor scheme for *Salmonella*^1^. The serotype is then expressed in the form of an antigenic formula, such as a list of factors O and factors H in the case of *E. coli* (e.g., *E coli* O157:H7). The scheme also provides a step-by-step procedure, which, by successive eliminations, leads to the single antigenic formula of the serotype (also called serovar). Many pathogenic bacteria have serotyping schemes more or less used by the scientific community according to their recognized discriminatory characteristics. The aforementioned White-Kauffmann-Le Minor scheme for *Salmonella* is an example of a serotyping scheme that is still a reference despite the advent of the more discriminatory (molecular) subtyping approaches. This scheme also has the particularity of associating a serotype name with a determined antigenic sequence. For instance, *Salmonella enterica* serotype Agona possesses the antigenic formula 1,4,[5],12:f,g,s:[1,2]. In contrast, no serotyping is available for foodborne viruses. Indeed, most of them (e.g., norovirus, hepatitis A virus, hepatitis E virus, etc.) are not cultivable *in vitro*, limiting the use of serum neutralization for typing purposes. In addition, no viral antigen detection tests are available for these viruses.

Antibiotyping involves testing the susceptibility of bacterial strains to different antibiotics. Their profiles of resistance/sensitivity to these antibiotics then defines the antibiotypes. If the list of molecules with antibiotic activity used is constant and for each molecule the concentration limit beyond which the bacterium is considered resistant is standardized by international or national organizations (i.e., EUCAST: European Committee on antimicrobial susceptibility testing), the antibiotype (or antibiotic resistance phenotype) becomes a characteristic (Threlfall et al., 1999) of a given strain and could contribute on works on source attribution (David et al., 2013a).

Phage typing is a phenotypic approach based on the sensitivity/resistance of a bacterium to bacteriophages. The method determines, among other things, the presence/absence of a bacterial receptor specific to each phage tested. The presence of this receptor is visualized by the appearance of lysis plaques on a culture of the bacterium in the presence of a suspension of the bacteriophage of adequate concentration. The method relies on a standard and stable panel of phages that determines the phage type (PT), and was developed for a limited number of bacterial species (Audurier et al., 1979) or subtypes (Anderson et al., 1977; Glosnicka and Dera-Tomaszewska, 1999).

Current developments in phenotyping methods are related to proteomics. The identification and analysis of polypeptide spectra expressed by a microorganism using MALDI-TOF (Matrix-Assisted Laser Desorption Time-Of-Flight) Mass Spectrometry is a good example of this novelty. These spectra, consisting of several hundred polypeptides expressed by the microorganism, can be exploited for identification of the genus and/or species or, under certain conditions, for the comparison of strains and also antibiotyping. The stability of the spectra according to the culture conditions must be verified beforehand. These methods are booming in diagnostic laboratories and are replacing classical phenotypical methods. Rapid improvement of these methods and their ability to provide cost-effective, high-quality data, highlight their relevance for future application in source attribution (Shell et al., 2017).

#### PCR-Based Methods

The first genotyping approaches were based on the amplification of genome fragments (genes or gene fragments, or even non-coding regions) by endpoint or real-time Polymerase Chain Reaction (PCR or qPCR) or Reverse Transcription PCR (RT-PCR). The analysis of this amplification is performed either directly by detecting the presence or absence of the expected gene fragment(s) or indirectly after digestion of the latter by an appropriate restriction enzyme. The selected restriction enzymes target short and frequently occurring cleavage sites on the genome. Under these conditions, a limited number of small-sized fragments are produced and these can be easily separated on an electrophoresis gel. The RFLP thus becomes a characteristic of the microorganism analyzed. For example, the sequences encoding 16S RNA in bacteria can be chosen (this is the principle of ribotyping) (De Cesare et al., 2001a, b) or more specifically, they are genes differing within a given bacterium that can be chosen to support this PCR-RFLP (e.g., *fla*-typing of *Campylobacter fla* gene, or *invA* or *fliC* for *Salmonella*) (Djordjevic et al., 2007). The choice of the gene and restriction enzymes used will influence the discriminatory power of the technique (Soler-Garcia et al., 2014). An alternative is the AFLP (Amplified fragment length polymorphism), which is based on the prior restriction of the genome and then the amplification of the produced fragments (Wang et al., 2011). PCR could also be used for bacterial genoserotyping, so by replacing the classical serotyping by a molecular serotyping for *Listeria monocytogenes* (Doumith et al., 2004). In the case of foodborne viruses, the reference detection method is real-time RT-PCR (RT-qPCR) with a specific probe or even digital PCR (dPCR). This approach may allow for a first level of typing with group-specific probes (e.g., norovirus Genogroup I or II).

Another approach consists of performing non-specific amplifications (degenerate primers and/or sub-stringent PCR conditions) in order to obtain, from the entire genome, randomly amplified DNA fragments that are variable but compatible with a migration in agarose gel. Under these conditions, the PCR generates a limited and reproducible number of fragments in a given laboratory. Their migration in a conventional agarose gel reveals the fragment size polymorphism (Random Amplified Polymorphic DNA or RAPD) for a microorganism. Different RAPD profiles then identify different strains. This approach has significant limitations regarding reproducibility and its value in comparing large microbial populations over time is rather low.

Another technique based on PCR is MLVA (Multi-Locus Variable Number Tandem repeat). Its principle is the following: in the genomes of bacteria and parasites, some sequences are repeated in tandem a certain number of times (Variable Number Tandem Repeat or VNTR, minisatellites or microsatellites). For a given VNTR, the size of the sequence is variable in multiples of the repeated unit. In addition, several VNTR zones of different compositions may be present in a genome. It is then possible to assess the diversity of the VNTR loci and, for each VNTR zone, the size of the amplified fragments, either by PCR and gel or by capillary sequencing or directly by analyzing the sequence. The standardization of this MLVA approach requires determining the number of VNTR loci retained in the analysis and considering the number of repeats found at each locus (Ferrari et al., 2017).

#### Methods Based on the Analysis of Restriction Fragments Length Polymorphism (RFLP)

The analysis of RFLP of the entire genome by Pulsed-Field Gel Electrophoresis (PFGE) is probably the one that benefited the most from developments before the arrival of high-throughput sequencing. The principle is to fragment a genome (from a bacterial culture) by restriction enzymes that target rarely represented cleavage sites. This results in a relatively limited number of fragments. Since the latter are large, they must be separated by specific electrophoresis, allowing for their migration. The development of this approach requires, for each bacterium, to select a set of enzyme (a single or several, i.e., *Asc*I/*Apa*I enzymes for *L. monocytogenes*) capable of cutting the genome into more or less 15 fragments of variable sizes for different strains (Graves and Swaminathan, 2001). The discriminatory power can be increased by using, in parallel, several restriction enzymes. This principle of digestion can also be applied not only to the genome, but on the plasmids extracted from bacteria (the enzymes are then chosen to generate fragments of size compatible with the normal agarose gel migration conditions). In doing so, one can obtain a plasmid profile for each bacterium. Thus, before comparing strains from a source attribution perspective, a protocol must be precisely defined, including the number and nature of the enzymes as well as the migration device used. This technique is robust and reproducible from one laboratory to another, but it may prove ineffective in discriminating closely related strains, i.e., strains belonging to the same species, originating from the same individual and having limited evolution from a genetic standpoint. This approach, which until recently has been recognized as a reference for many bacteria, is being now replaced by sequencing-based approaches.

#### Methods Based on the Analysis of Genomic Sequences

Multi-Locus Sequence Typing (MLST) relies on the presence of allelic diversity at a certain number of loci in the genome of a bacterial species. After sequencing each locus, an allele number is determined, with the combination of these numbers defining the sequence type (ST) and clonal complexes (CC). Before relying on an MLST method to compare strains, a consensus must be reached on the number and nature of the loci to sequence for ST identification. There is also a need for a large ST bank (in terms of number and diversity) to be established before defining the discriminatory power of this method for a given microorganism. For viruses, the block fragments are chosen in a genomic region with discriminatory power. This genomic region must be framed by sequences sufficiently conserved to be amplified using consensus primers and must contain sufficient variability to differentiate the different subtypes or subgroups.

The CRISPR-Cas system is considered to be a prokaryotic immune system based on the integration of bacteria-specific nucleotide sequences in specific regions of the genome: CRISPR (Clustered Regularly Interspaced Short Palindromic Repeats). These regions are reorganized over time by bacteria (new spacer acquisitions, depletion of obsolete ones). Therefore, the sequencing of these zones of the genome makes it possible to differentiate the strains and even to infer their phylogeny.

The latest developments in molecular methods are based on preliminary comparative results of whole genomes. This comparison establishes a list of genes whose presence/absence is frequent enough to distribute the population of the tested bacteria. This technique is particularly adapted to genomes showing great plasticity. In *Campylobacter*, this CGF (Comparative Genomic Fingerprinting) technique is based on the detection of 40 genes (by a reasonable number of multiplex PCRs) and produces a presence/absence code for these 40 genes: the typical CGF. The CGF technique delivers consistent results with the MLST scheme and provides a higher level of discrimination among strains (Ravel et al., 2017). This approach is also often called haplotyping, expressly developed for *Salmonella* Typhi (>80 genes).

Whole Genome Sequencing (WGS) is enabled by high-throughput sequencing techniques. The benefit is then the availability of complete genomes to be compared. Given the huge amount of data provided simultaneously by WGS, the challenges of analyzing the sequences, as well as to define their quality criteria and to obtain alignment, require advanced bioinformatics skills. The harmonization of these bioinformatics ‘pipelines’ is the key to standardization of these approaches. Sequencing of complete genomes allows for the number and position of single nucleotide polymorphisms (SNPs) to be identified and differentiated among strains, including bacterial species previously considered to be highly homogeneous from a genetic point of view. An ultimate level of discrimination among close strains is reached. The availability of these sequences makes it possible to select those that are relevant for the comparison (or phylogeny) of the strains. The application of MLST approaches to the core genome (cgMLST) or whole-genome genes (wgMLST) is an example of the development of highly discriminatory typing methods derived from WGS approaches applicable to source attribution (Sheppard et al., 2012). For example, if MLST would compare 5–7 loci, the cgMLST would be based on the comparison of several hundreds or thousands of genes or variable sequences. For example, cgMLST for *L. monocytogenes* allows for rapid analysis of sequences based on the comparison of allele numbers and their sum up in a cgMLST type (CT) with a number (e.g., CT577) that could be easily exchanged among laboratories, food business operators and risk managers (Moura et al., 2016).

### Standardization, Automation, and Discriminatory Power of Typing Methods

For the purpose of source attribution, a suitable subtyping method must essentially satisfy the following three conditions (Table 1): (1) be standardized in order to facilitate the exchange of data and the comparison of results among laboratories; (2) be automated with a reference data set allowing for the establishment of a nomenclature within the microbial species (e.g., serotyping, MLST, PFGE, cgMLST, etc.); (3) be discriminatory at the level of the individual microorganism.

**TABLE 1:** Typing methods and their discriminatory power, level of automation and standardization.

**Method type**	**Method name**	**Discriminatory power**	**Automatization**	**Standardization**
Phenotyping	Speciation-biotyping	Low	Yes	Yes
	Antibiotyping	Low	Partially	Yes
Agglutination serum	Serotyping	Low to high	No	Yes
Lysotype	Lysotyping	Moderate	No	Yes
Maldi-Tof MS spectra	Maldi-TOF (mass spectometry)	Low to moderate	Yes	Yes
	Phagetyping	Moderate	No	Yes
DNA macrorestriction on gel	Ribotyping	Moderate	Partially	Yes
	Plasmid profiling	Low	No	No
	AFLP	Moderate	No	No
	RAPD	Moderate	No	No
	IS200^1^	Moderate	No	No
	PFGE	Moderate to high	No	Yes
Nucleotide targets	Targeted sequencing	High	Yes	Yes
	MLST	Moderate to high	Yes	Yes
	MLVA	High	Yes	Yes
	CRISPR	Moderate	Yes	No
	Real-time RT-qPCR	High	Yes	No
	WGS (cgMLST, wgMLST, SNP)	High	Yes	No

*^1^IS 200 Amplification by PCR of an insertion sequence (Threlfall et al., 1994).*

Standardization and validation of a typing method are the result of a consensus by the scientific community on a common protocol of analysis, providing comparable data based on an inter-laboratory study and a satisfactory level of discrimination in terms of diversity among strains. It may be a serotyping scheme (e.g., White-Kauffman-Le Minor for *Salmonella*), the nature and number of restriction enzymes necessary to establish a PFGE profile for *L. monocytogenes*, or the nature and number of sequences to be analyzed for characterizing a *Campylobacter* ST. Yet, this consensus is not always reached. This is true both for phenotyping approaches (e.g., for *Salmonella*, several different phage typing systems have long existed in parallel) and for MLST approaches (i.e., the number of genes analyzed to define the ST differs among laboratories). Sometimes standardization is impossible because the results are not that reproducible, such as in the case of RAPD. As several laboratories are nowadays engaged in the characterization of strains, there is a needed for standardization of typing methods and assessment of performance in inter-comparison tests before pooling results from different origins (e.g., the PulseNet International Protocol)^2^. For WGS, a working group ISO TC34/SC9/WG25 at ISO (International Organization for standardization) developed an international standard on WGS, typing and genomic characterization of foodborne bacteria.

For noroviruses and hepatitis A virus, there is a standard established by the European Committee for Standardization (CEN) and internationally validated on horizontal methods of analysis of foodstuffs (EN ISO15216-1 and CEN ISO/TS 15216-2). These methods defining the conditions for viral detection or quantification in food matrices are based on RT-qPCR and allow for discrimination among genogroups GI and GII in specific foods. For parasites, there is no standardized detection method for *Toxoplasma gondii* in meat or vegetables, but when the parasite is isolated, genotyping methods can be applied (e.g., microlens, MLVA, RFLP). The complete sequencing technique is being developed (34 strains of *T. gondii* have been fully sequenced to date). For *Cryptosporidium* and *Giardia duodenalis*, there is a standardized method for detection in water (NF T 90-455), fresh leafy green vegetables and berries (EN ISO 18744), without characterization or genotyping. For strains from human or animal reservoirs, characterization is genotypic and relies on microsatellite MLVA typing or on the analysis of the sequence diversity of regions amplified by PCR or qPCR (Table 2).

**TABLE 2:** Typing techniques of reference, those routinely used and those most discriminatory per each foodborne pathogen.

**Pathogen**	**Reference technique**	**Standardized techniques in common practice**	**Most discriminatory technique**
*Bacillus cereus*	Genotyping, toxins and toxin genes	Genotyping	WGS^2^
*Clostridium perfringens*	Toxins and toxin genes	Toxinotyping^1^	WGS
*Campylobacter* spp.	Biochemistry and MLST	Biochemistry and MLST	WGS
STEC	Serotyping, toxin profiling	Serotyping, toxin profiling,^2^ PFGE	WGS
*Listeria monocytogenes*	Genoserotyping, MLST, cgMLST, SNPs	Genotyping, PFGE (PulseNet protocol), AFLP (UK), MLST, cgMLST, wgMLST, SNPs	cgMLST, SNPs
*Salmonella* spp.	Serotyping (White-Kauffmann-Le Minor), PFGE (PulseNet protocol)	Serotyping, PFGE, MLST, MLVA (Typhimurium and Enteritidis), WGS	WGS
*Shigella* spp.	Serotyping and biotyping	Serotyping, biotyping, MLVA	WGS
*Staphylococcus aureus*	Serotyping	Serotyping, PFGE, spa typing, MLST	WGS
*Yersinia enterocolitica*	Biotyping, serotyping, PFGE	Biotyping, serotyping, PFGE, SNPs, cgMLST	WGS
Norovirus	Real-time or conventional RT-PCR + sequencing, genogrouping and genotyping	Genogrouping and genotyping	Genogrouping
HAV	Real-time or conventional RT-PCR + sequencing, genogrouping and genotyping	Genogrouping and genotyping	Genotyping
HEV	Real-time or conventional RT-PCR + sequencing, genogrouping and genotyping	Genotyping and subtypes	Genotyping and subtypes
*Cryptosporidium* spp.	Real-Time PCR (ARN 18S) and PCR of the microsatellites *gp60* (for all species) and *Cp47* (for *C. parvum*) and then sequencing	Real-time PCR (RNA 18S) and PCR of the microsatellites *gp60* (for all species) and *Cp47* (for *C. parvum*) and then sequencing	Microsatellites *gp60, Cp47, RPGR, MSC6-7*
*Giardia duodenalis*	PCR of the *tpi* (triose phosphate isomerase) gene and of the β*-giardine* gene and then sequencing	PCR of the *tpi* (triose phosphate isomerase) gene and of the β*-giardine* gene and then sequencing	Sequencing β*-giardine* + *tpi*
*Toxoplasma gondii*	Microsatellite genotyping and RFLP	Microsatellites and RFLP	Microsatellites

*^1^Typing of toxins; ^2^Pathogenicity genes: *eae*, *ehxA*, *stx* variants and WGS methods: SNPs, cgMLST, wgMLST.*

Automation is rarely complete. The most recent techniques incorporate certain automated steps, such as DNA or RNA extraction, validation of process quality including sequencing, and use of pipelines for bioinformatics. Currently available typing approaches still require some fundamental manual activity to varying degrees. It should also be considered that these typing steps are preceded by detection methods, which require direct interventions and specific ‘know-hows’ as well. For example, the search for viruses in non-vegetable matrices may require dissection procedures for digestive tissues of mollusks or grinding of complex meat-based matrices (e.g., degreased sausages), but the automation of these procedures is not often foreseen.

Discriminatory power is not a performance characteristic of a given method but the product of the method and microorganism in question (including the set of strains studied). The most striking example is the passage of two to three restriction enzymes for *S*. Enteritidis pulsotyping when two are sufficient to differentiate PFGE profiles from *S*. Typhimurium. Similarly, if an MLST is unable to differentiate two epidemiologically distant strains, the addition of loci to be sequenced (toward cgMLST or haplotyping) can overcome this difficulty. But these adjustments have to be made *a priori* and cannot be considered as possible adjustments during the analysis. The discriminatory power is therefore not a limit, especially when dealing with sequences of the complete genome.

### Source Attribution Models Based on Microbial Subtyping

The principle of source attribution based on microbial subtyping is to attribute human cases caused by a given pathogen to a number of putative sources of infection based on the distribution of pathogen subtypes in humans and sources. The subtypes are usually defined by phenotyping (namely serotyping, phage typing, and antimicrobial profiling, e.g., for *Salmonella*) and/or genotyping methods (e.g., cgMLST for *Listeria monocytogenes* and *Campylobacter*) (Sheppard et al., 2012; Taboada et al., 2012; Ruppitsch et al., 2015; Moura et al., 2016; Thepault et al., 2017). The use of standardized subtyping methods is then a prerequisite (e.g., PulseNet International, a laboratory network dedicated to tracking foodborne infections worldwide by promoting standards for PFGE for molecular typing of foodborne pathogens), as is the optimal level of discrimination of the typing method in question. Of note, these methods are particularly demanding in terms of data requirements and computational capacity. For source attribution, besides subtyping data *per se*, it is also crucial to consider the representativeness of strain collections. Inclusion of data on the level of contamination (i.e., pathogen prevalence/concentration) of the sources, as well as data on the degree of exposure of the population to these sources (e.g., amount of food consumed) are also sometimes necessary to scale the similarities in pathogen subtypes between humans and sources.

The optimal discrimination power of the subtyping method depends on the source attribution method applied. The subtypes or groups of subtypes should be chosen to address the need to have groups large enough to find matches between cases and sources, but not too large to penalize precision in the attribution results. Alternatively, some source attribution models may use phylogenic relations among strains, accounting for their potential evolution by, e.g., explicitly addressing mutation and recombination events.

Source attribution methods based on microbial subtyping attribute cases to *a priori* chosen sources. Consequently, all main potential sources must be included in the analysis, with a representative and heterogeneous distribution of subtypes among these sources. Travel-related cases need also to be identified and excluded from the source attribution analysis, as they are not due to exposure to domestically available foods. Only one case per outbreak event is usually included in the analysis to avoid inflating attributions to a given food source because of, e.g., poor hygiene practices of food preparation and consumption, and not because the actual level of contamination of the source itself. Moreover, excluding outbreak-related cases from the source attribution analysis (of sporadic cases) would avoid over-representing those cases attributable to a specific source due to increased testing in response to public alert following an outbreak event receiving considerable media attention. Examples are the large *Salmonella* Thompson outbreak linked to smoked salmon in the Netherlands in 2012 leading to over 1100 cases identified (Friesema et al., 2014), or the German outbreak of *E. coli* O104:H4 linked to sprouts where over 4000 cases were reported (Buchholz et al., 2011).

#### Frequency-Matching Models

**FIGURE 2:**
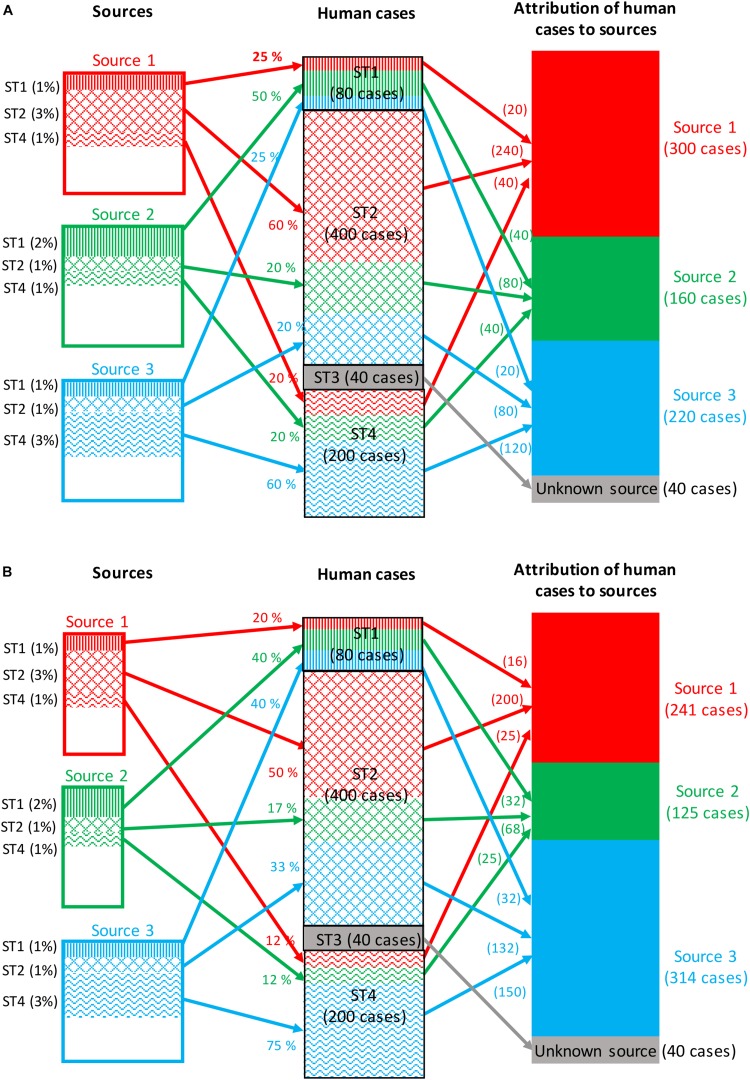
Example of attribution of human cases (720 cases) of a given foodborne disease to three potential sources based on four microbial subtypes. **(A)** The attribution takes into account only the prevalence of all subtypes in each source, and the exposure to each source is then assumed to be constant. **(B)** The attribution takes into account both the prevalence and the exposure to each source.

Frequency-matching models for source attribution infer probabilistically the most likely sources of human cases by comparing their subtype frequencies, weighted by factors like prevalence in these sources and the human exposure to them. Frequency-matching models therefore assume that the subtypes remain stable when passing from their (food, animal, or environmental) sources to humans. A measure of the area of overlap between the subtypes frequency distributions of human and source strains can be computed using the Proportional Similarity Index (PSI) or Czekanowski Index (Feinsinger and Spears, 1981; Rosef et al., 1985). Figure 2A illustrates a situation in which all human cases are associated with four subtypes of a given pathogen: 80 cases associated with subtype 1, 400 cases with subtype 2, 40 cases with subtype 3, and 200 cases with subtype 4. The frequencies of detection of these four subtypes in the three sources considered are:

• Subtype 1: 1, 2, and 1% for sources 1, 2, and 3, respectively.
• Subtype 2: 3, 1, and 1% for sources 1, 2, and 3, respectively.
• Subtype 3: absence of isolation in all three sources.
• Subtype 4: 1, 1, and 3% for sources 1, 2, and 3, respectively.

Assuming that the exposure to the three sources is the same, the calculation of the attributable fractions for, e.g., subtype 1, would be 25% [1/(1 + 2 + 1)], 50% [2/(1 + 2 + 1)] and 25% [1/(1 + 2 + 1)] for sources 1, 2, and 3, respectively. By applying these percentages to the number of human cases caused by subtype 1 (i.e., 80 cases), it can be calculated that 20, 40, and 20 cases are attributable to sources 1, 2, and 3, respectively. This calculation is then repeated for all subtypes, and a summation over subtypes gives the total number of cases attributed to each source.

As mentioned before, the subtype frequencies can also be weighted by factors like the level of exposure to sources and the ability of the different subtypes to cause infection (e.g., pathogenicity). Figure 2B illustrates the same calculation by integrating a weight reflecting these factors for each source. The population now appears to be twice as likely to be exposed to source 3; thus, the weighted frequencies now lead to 20, 40, and 40% attributions of subtype 1 to sources 1, 2, and 3, respectively.

The results of a source attribution analysis are usually presented as the number, or percentage, of human cases attributed to each source. These estimates are often accompanied by confidence intervals or, more commonly, credibility intervals when Bayesian models are used. A temporal dimension for source attribution has also been proposed (Mullner et al., 2009a; Pires and Hald, 2010; Ranta et al., 2011; Mughini-Gras et al., 2014a). The frequency-matching models commonly used in the literature are the Hald (‘Danish’) and the Dutch models.

##### The Hald (‘Danish’) model and its adaptations

Hald et al. (2004) proposed a Bayesian model for source attribution of non-typhoid salmonellosis in Denmark based on serotyping and phage typing data. The model is written as:

$$o_{i}∼\text{P}\,\mathrm{oisson}\,\left({\text{Σ}}_{j}\,\lambda_{i\,j}\right)\;w\,i\,t\,h\,\lambda_{i\,j}=p_{i\,j}\times M_{j}\times q_{i}\times a_{j}$$

where,

*i*: Index of subtypes, varying from *1* to *I*,
*j*: Index of sources, varying from *1* to *J*,
Data:
*o*_*i*_: Observed number of human cases caused by subtype *i*,
*p*_*ij*_: Prevalence of subtype *i* in source *j*,
*M*_*j*_: Amount of food from source *j* consumed by the population.

Parameters to be estimated:

λ_*ij*_: Expected number of human cases of subtype *i* from source *j*,
*a*_*j*_: Source-dependent parameter,
*q*_*i*_: Subtype-dependent parameter.

The Hald model therefore takes into account the subtype prevalence in each source (*p*_*ij*_), the amount of food consumed from each source (*M*_*j*_), the ability of each source to act as a vehicle for the pathogen in question (*a*_*j*_), and the ability (e.g., pathogenicity, infectious dose, fitness, etc.) to cause infection of each subtype (*q*_*i*_). As an example, the average number of human *S. enterica* serotype Heidelberg infections deriving from consumption of chicken would be proportional to the prevalence of *S*. Heidelberg in chicken, the amount of chicken consumed by the population, the ability of chicken meat to allow for *S. enterica* transmission to humans, and the ability of *S*. Heidelberg to cause infection in humans. While *p*_*ij*_ and *M*_*j*_are based on known values, *a*_*j*_ and *q*_*i*_ are unknown and are, thus, estimated by the model.

Parameter estimation is based on Bayesian inference, including both informative and non-informative priors. However, the model is over-parameterized, i.e., there are fewer observations than parameters to be estimated. To circumvent this issue, some parameters were fixed to the same values in the original Hald model. For instance, the subtype-dependent parameter (*q*_*i*_) was assumed to be identical for all phage types within the two *S. enterica* serotypes Typhimurium and Enteritidis (most frequent types in humans) and, for the most common serotype (*S*. Enteritidis), this value was arbitrarily set to 1. Fixing parameters does improve model identifiability (i.e., the true values of the model’s underlying parameters can be theoretically obtained from an infinite number of observations), but it does so at the expense of an artificial reduction of the uncertainty in the estimates. Moreover, such arbitrary choices may have important consequences in the attribution results (David et al., 2013a). The presence of specific subtypes in sufficient number (at least as much as the sources) allows arbitrary assumptions to be lifted using values calculated from the data themselves (David et al., 2013a).

Another approach allowing the model to become more identifiable consists of parameterizing the subtype-dependent factors (*q*_*i*_) as random effects from a non-informative *a priori* distribution where only the first two moments (hyper-parameters) of the distribution are estimated (Mullner et al., 2009a). For example, in the case of a Gaussian distribution, the first two moments would correspond to the mean and variance. Although the type-dependent parameters are no longer estimated, they can be predicted if necessary.

Several modifications have been proposed to adapt the Hald model to pathogens other than *Salmonella*, as well as to different epidemiological contexts and data types, including the integration of non-food sources (Mullner et al., 2009a; Mughini-Gras et al., 2016, 2019), use of passive surveillance data (David et al., 2013b; Glass et al., 2016), a combination of source data from different monitoring systems (Mullner et al., 2009a), non-availability of exposure data (Mullner et al., 2009a; Mughini-Gras and van Pelt, 2014; Glass et al., 2016), different inclusion modalities for outbreak data (Glass et al., 2016), inclusion of both epidemiological and genetic data (Liao et al., 2019), and presence of sparse data (Mikkela et al., 2019). Of these model adaptations, the most successful one is probably the ‘modified Hald model’ (Mullner et al., 2009a), which besides removing parameter (*M*_*j*_) to include also non-food sources (as this effect will be absorbed by *a*_*j*_), it includes a modeled subtype-specific prevalence (*p*_*ij*_) derived from the product of the within-source subtype relative frequencies and the overall prevalence of the pathogen in question in each source.

Recently, Miller et al. (2017) developed a novel source attribution model, named ‘sourceR’, which builds upon, and unites, the original Hald (Hald et al., 2004) and modified Hald (Mullner et al., 2009a) models. The model is flexible, fully joint (as it combined the different outcomes in a single model), and does not rely on many approximations and assumptions. Mixing and *a posteriori* correlations are significantly decreased in comparison to the modified Hald model. Moreover, a Bayesian non-parametric model (Dirichlet process) is used to inform strain-dependent clustering effects, allowing for the identification of strain clusters with similar virulence, pathogenicity, and survivability. This is a significant enhancement, allowing for identifiability improvement over the previous models (i.e., fixing some parameters *a priori* or modeling the type effects hierarchically as random effects). The model also incorporates uncertainty in the prevalence, but it does so by fitting a fully joint model rather than a two-step model like in the modified Hald model (Mullner et al., 2009a). This has the advantage of allowing the human cases to influence the uncertainty in the source data and to preserve the restriction on the sum of the prevalences for each source (Miller et al., 2017).

##### The Dutch model and its adaptations

The frequentist model proposed by van Pelt et al. (1999) allows for direct (proportional) source attribution of non-typhoid *Salmonella* (the so-called “Dutch” model). Using previous notations, the model can be written as follows:

$$\lambda_{i\,j}=\hat{P}\,\left(s\,o\,u\,r\,c\,e\,j|s\,u\,b\,t\,y\,p\,e\,i\right)\times o_{i}$$

In this model, the number of human cases of subtype *i* attributable to source *j* is proportional to the observed number of human cases of subtype *i* multiplied by the probability for this subtype *i* of coming from source *j*. In the original Dutch model, this probability is directly estimated from the frequencies of the subtypes in the sources (*r*_*ij*_):

$$\hat{P}\,\left(s\,o\,u\,r\,c\,e\,j|s\,u\,b\,t\,y\,p\,e\,i\right)=\frac{r_{i\,j}}{\sum_{j}r_{i\,j}}$$

This model does not take into account differences among sources (e.g., exposure) or among subtypes (e.g., pathogenicity) in their ability to cause infection, thereby assuming an equal impact of the different sources and subtypes on the human population. An illustration of the Dutch model is presented in Figure 2A. In subsequent model adaptations, the probability was estimated using the approach proposed by Mullner et al. (2009a) and food consumption weights were also included to take into account differences in exposure to different sources (Ranta et al., 2011; Mughini-Gras et al., 2014a, b). However, attributions appear to be very sensitive to changes in these food consumption weights. Consequently, an additional parameter denoting the probability for these foods to be consumed raw/undercooked by the population has been proposed (Mughini-Gras et al., 2014b; Mughini-Gras and van Pelt, 2014). Such probability essentially reflects the ability of the sources to act as a vehicle for the pathogen in question, which lifts the assumption of equal impact of the different sources on the human population.

##### Generalities of the Hald and Dutch models

When confidence in data is low, some authors suggest introducing uncertainty and additional information into the (modified) Hald and Dutch models, particularly in the prevalence and food consumption weights (Mullner et al., 2009a; Mughini-Gras and van Pelt, 2014). In situations where information about human cases (e.g., subtyping of pathogens, travel status of cases, etc.) is partially missing, Hald et al. (2004) and de Knegt et al. (2015) proposed to reassign these cases according to the observed distribution of subtypes and known statuses of cases, and this has been done by others as well (Mughini-Gras et al., 2014a). Moreover, when the prevalence of the pathogen in a source is unknown, Mullner et al. (2009a) propose a few methods to combine surveillance data with other data approximating prevalence. Working with time series, Ranta et al. (2011) proposed a non-parametric model to integrate the sources when data are available only at certain period.

Being quite data-intensive and computationally demanding, the modifications that have been proposed to mitigate some of the limitations of the original Hald model are mainly applied nowadays, with novel applications like sourceR enabling more straightforward attributions to be estimated (Miller et al., 2017). Several modifications to the Dutch model have also been proposed to mitigate some of its questionable assumptions, such as the assumed equal impact of sources on humans (Mughini-Gras et al., 2014a,b,c, Mughini-Gras and van Pelt, 2014). These modifications have been mostly based on the more recent developments of the (modified) Hald model. Notably, of the frequentist framework of the original Dutch model for direct attribution, its modified versions go toward a more stochastic framework based on Monte Carlo simulation.

The (modified) Dutch and Hald models have been extensively used for source attribution of major (bacterial) foodborne pathogens. Studies have focused on *Salmonella* (Hald et al., 2004, 2007; Mullner et al., 2009a; Pires and Hald, 2010; Guo et al., 2011; Wahlstrom et al., 2011; David et al., 2013a, b; Mughini-Gras et al., 2014a,b,c, 2016; Mughini-Gras and van Pelt, 2014; de Knegt et al., 2015, 2016; Vieira et al., 2016; Ahlstrom et al., 2017; Mikkela et al., 2019) and *Campylobacter* (Mullner et al., 2009a, b; Ranta et al., 2011; Boysen et al., 2014; Liao et al., 2019), and to a lesser extent on *L. monocytogenes* (Little et al., 2010; Nielsen et al., 2017), and *Shiga*-toxin producing *E. coli* (STEC) (Mughini-Gras et al., 2018b).

An important limitation of both the Dutch and Hald models is that they do not allow for the attribution of microbial subtypes identified in humans but not in the sources, thus generating a fraction of non-attributable cases or cases of ‘unknown source’. Moreover, human cases of infections with subtypes present in the sources will be attributed to these sources even if they are actually linked to other sources (e.g., not considered in the model), thereby the importance of including as many sources as possible. It has been shown that including sources considered of minor importance could lead to the reassignment of 25% of the cases initially attributed to known sources of *Salmonella* (David et al., 2013b). These models also assume that the different subtypes are independent of the sources, whereas biological interactions between specific pathogens and foods exist. In addition, no model satisfies all needs and several models are often used in a comparative fashion (Mullner et al., 2009a, b; Mughini-Gras et al., 2014a, c, 2018b; Mughini-Gras and van Pelt, 2014).

#### Population Genetics Models

Genetic variations in microorganisms are the result of different evolutionary forces. These can be prompted by either neutral processes (genetic drift) or adaptive processes, such as the emergence of a competitively advantageous mutation in a given environment. Most bacterial populations are structured, i.e., they do not form a genetically homogeneous unit, but rather consist of several distinct lineages that are totally or partially isolated from one another. Geographical isolation, combined with random phenomena of genetic drift and sometimes with local adaptation, drives genetic differentiation.

When analyzing genetic data for a given microorganism based on targets like a certain number of alleles, microsatellites, or SNPs, the objective is often to detect whether these microorganisms are structured into different subpopulations, and if so, to identify the number of clusters, the strains composing them, and possibly their recombination events (phylogeny). Historical methods for studying the genetic proximity of microbial populations are based on the construction of phylogenetic trees from a matrix of proximities for each pair of strains, with these genetic proximities being typically calculated using the methods proposed by Nei et al. (1983) or Reynolds et al. (1983). Once the tree is built, it can be ‘cut’ at a certain point (e.g., after three levels of nodes from the root) to define the different clusters (more or less equivalent to subtypes). Another approach is to assume that genetic data (e.g., frequency of different allele numbers at a locus) can be explained by a probabilistic model whose parameters are unknown. Comparing genetic data (frequencies) among different strain populations allows us to establish a link between them, e.g., between strains from human cases and from different sources. Two population genetics models that are currently widely used for source attribution of foodborne diseases are the so-called STRUCTURE (Pritchard et al., 2000) model and the Asymmetric Island Model (AIM) (Wilson et al., 2008). These two models are based on different principles of genetic structuring of microbial populations, but the overall attribution approach is similar.

##### STRUCTURE

STRUCTURE has been developed by Pritchard et al. (2000) and is one of the first explicit models examining the genetic structure of microbial populations. This model assumes the existence of *K* (unknown) populations, each of which is characterized by a set of allelic frequencies at each genetic locus considered. In the simplest model without admixture, each strain is attributed to a single population. The probabilities that a strain belongs to the other populations reflect the attribution uncertainty. In the model with admixture, each locus of a strain is attributed to a population: a strain can therefore be attributed jointly to several populations.

**FIGURE 3:**
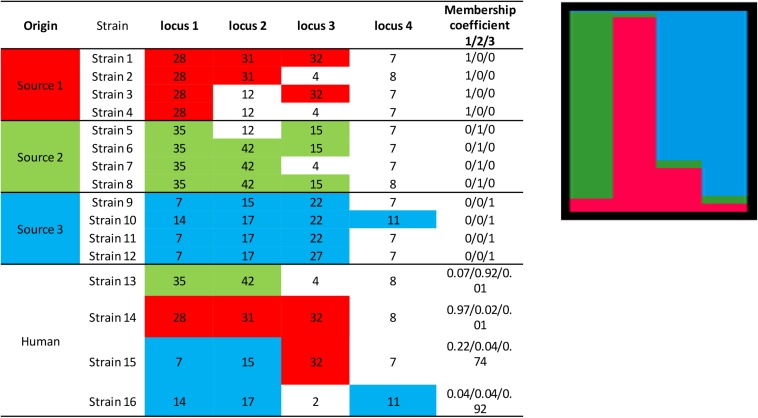
Illustration of the approach for source attribution of the STRUCTURE model. Table: allelic profile of 12 strains from three sources (source 1 in red, source 2 in green, source 3 in blue) and four human strains. Bar chart: membership coefficients of the four human strains for the three sources. Each vertical bar represents a strain to be assigned. The relative lengths of the color bars for a strain are proportional to the membership coefficients.

The principle of the model is to estimate the allelic frequencies in different populations and their admixtures using Bayesian inference. Tracing the sources of human cases is a particular case of this model without admixture of the source strains, that is, the strains can only belong to one of the *K* populations, each of which corresponds to a specific source. The allelic frequencies at each locus are characterized for each of the *K* populations and the strains to be attributed are established from frequencies of characteristic allelic numbers at each locus. Using a fictitious data set, Figure 3 illustrates the STRUCTURE approach, including the calculation of the membership coefficients for the different sources. In this example, 12 strains (from three different sources) are characterized by a given allelic number at four loci. The alleles in colored boxes are those specifically detected (or present) in a given source. The membership coefficients of the strains are calculated from the allelic frequencies in the three sources. This means that, e.g., for strain 13, the allelic number 35 at locus 1 is very specific to source 2 (present in 100% of the strains of that source), so it weighs heavily in the calculation of the membership coefficient. The allelic number 42 of locus 2 is also specific to source 2 (but only 75% of the strains of this source have it). The allelic numbers 4 and 8 of loci 3 and 4, respectively, are present in sources 1 and 2 and absent in source 3. This explains why the probability for strain 13 to belong to this latter source is very low.

STRUCTURE can be applied to different genetic targets, such as microsatellites, MLST, RFLPs, AFLP, SNPs, etc. provided that this information is available for different loci. It is also necessary that common alleles show some intrinsic diversity. In theory, this approach could work with information limited to two loci and two variants per locus. In practice, however, data often derive from subtyping methods that consider several loci and variants (see section “Genotyping methods”). Most publications using this approach attributed human cases to specific reservoirs (Sheppard et al., 2009; Strachan et al., 2009; Kittl et al., 2013; Roux et al., 2013), farms (Müllner et al., 2010), or sources in general (Nielsen et al., 2017). The results are mostly presented in graphical form like in Figure 3, also for individual strains, or as percent attributions, corresponding to the average of the membership coefficients. The overall attributions are sometimes accompanied by measures of uncertainty like 95% confidence or credibility intervals.

The model is available as an open-access software and its main advantage is that it allows for several loci to be considered, providing the opportunity for use of whole-genome sequencing (WGS) derived data. The main recognized limitation of this model is related to the definition of the optimal number of populations (*K*). However, in the specific case of source attribution, *K* corresponds to the number of sources, and because of non-admixture, a strain can only originate from one source.

##### Asymmetric island model (AIM)

This model was developed by Wilson et al. (2008) for *Campylobacter* and, in its original formulation, it aims to infer the population structure, as well as to explain the genetic differentiation through phenomena of mutation, recombination and migration, as an extension of Wright’s island model (Bodmer and Cavalli-Sforza, 1968). In this model, the population is separated into different islands. After a number of generations, individuals migrate among these islands. The level of genetic differentiation among populations is therefore a function of the number of migrants in each island at each generation. The extension of this model allows for different, i.e., asymmetric, migration rates. In the framework of source attribution, the population to be attributed corresponds to the strains from human cases, and the different islands correspond to their different sources. Based on the allelic frequencies at given loci in a population of known sources, it is possible to attribute the origins of the human strains, as well as to estimate the mutation, recombination and migration rates. The rates of migration from the sources to the human population correspond to the parameters of interest, i.e., the attribution of human cases.

**FIGURE 4:**
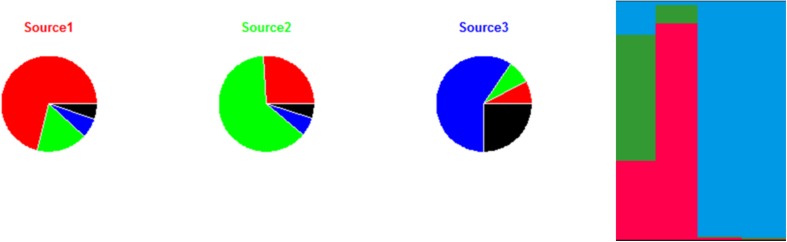
Illustration of the approach for source attribution of the asymmetric island model. Pie charts: migration rate (segments with colors different from the source name) and mutation (black segments) for each of the three sources according to the allelic frequencies of the sources shown in Figure 3. Bar chart: attribution probabilities of the four human strains for the three sources (source 1 in red, source 2 in green and source 3 in blue) estimated by the asymmetric island model according to the allelic profiles presented in Figure 3. Each vertical bar represents a strain. The relative lengths of the color bars for a strain are proportional to their attribution probability.

An illustration of the AIM based on the same data set used previously to illustrate the STRUCTURE approach (four strains to be attributed to three sources) is provided in Figure 4. Migration, mutation, and recombination rates are calculated from the alleles presented in Figure 3. Figure 4 shows the estimates for the migration and mutation rates: source 3 has the largest fraction associated with mutation in the same source (black segment), and this is explained by the appearance of the new allelic numbers 14, 15, and 27 at loci 1, 2, and 3. Sources 1 and 2 have higher migration rates than source 3, as they share the same allelic numbers (e.g., 12 at locus 2, and 4 at locus 3). Once mutation, recombination and migration rates are estimated, the attribution probabilities for the human strains can be calculated. Figure 4 shows these probabilities based on the fictional allelic profiles of Figure 3.

The AIM can be applied to different types of genetic markers, especially MLST and MLVA. The same data needs of STRUCTURE apply to the AIM. Most studies using the AIM concern *Campylobacter* (with 7-loci MLST as the most common target) (Wilson et al., 2008; Sheppard et al., 2009; Mughini Gras et al., 2012; Smid et al., 2013; Mossong et al., 2016), *S. enterica* (5-loci MLVA) (Mughini-Gras et al., 2014c; Barco et al., 2015), and *L. monocytogenes* (7-loci MLST) (Maury et al., 2016; Nielsen et al., 2017). A high degree of genetic diversity within the sources, low divergence among sources, and limited data are reported as the main limitations to the applicability of this model (Barco et al., 2015; Mughini-Gras et al., 2018a).

The assumptions of the AIM are stronger and closer to the biology of some foodborne pathogens than those of STRUCTURE. Indeed, the AIM also contemplates the possibility of non-independence between loci and accounts for migration of strains between sources, which is a biological reality. However, two main limitations can be identified: (1) the model attributes all human strains, even those that are not found in sources; (2) a new strain that has never been found in the sources is attributed to the source with the highest recombination rates.

##### Generalities of population genetics approaches

Population genetics models can be empirically validated using self-attribution (i.e., attribution of a random subset of sources strains) (Sheppard et al., 2009; Kittl et al., 2013), which provides an indication that the attributions are sound. Few comparative studies of STRUCTURE and the AIM have been published (Sheppard et al., 2009). It is, therefore, difficult to recommend the use of one model over another, even though the assumptions associated with the AIM appear to be closer to the evolution of clones within and between different sources. The results of the self-attribution are undoubtedly the best criterion for choosing a model. The results obtained by these two models are also often compared or supplemented by other approaches, including those based on indices of genetic proximity or diversity (e.g., Simpson’s index of diversity and analysis of molecular variance) (Excoffier et al., 2005; Sheppard et al., 2009; Kittl et al., 2013; Roux et al., 2013). Among phylogenetic approaches, recombination events are taken into account in order to improve the input data of these models (Didelot and Falush, 2007; Strachan et al., 2009; Roux et al., 2013), and new phylogeographic approaches have recently been proposed to complement source attribution (Dearlove et al., 2016). Comparison with epidemiological approaches (case-control and case-studies) (Roux et al., 2013) or results obtained from other population genetics models like BAPS (Bayesian Analysis of Population Structure) (Dale et al., 2011) are also available.

## Combined Epidemiological and Microbiological Methods

Case-control studies alone do not suffice to attribute human cases to reservoirs, as they can only trace back to the sources of human infections up to the point of exposure (e.g., foods consumed), which may not correspond to the original reservoirs because of, for instance, cross-contamination or alternative transmission routes. On the other hand, source attribution based on microbial subtyping allows to determine the relative contributions of different reservoirs to the human disease burden, i.e., to attribute the human cases up to the top of the transmission chain. Combining source attribution and case-control data therefore allows to reconstruct the underlying transmission pathway, from a given reservoir up to the point of exposure/risk factor, providing a more complete epidemiological picture than when performing separate analyses. To this aim, subtyping of strains included in case-control studies has been undertaken so that a combined source attribution and case-control analysis can be performed. This combined analysis is called “source-assigned case control study” and has been performed for *Campylobacter* in the Netherlands (Mughini Gras et al., 2012) Luxembourg (Mossong et al., 2016), Germany (Rosner et al., 2017), Scotland (Bessell et al., 2012), Canada (Levesque et al., 2013), and New Zealand (Mullner et al., 2010), as well as for *S. enterica* (Mughini-Gras et al., 2014b) and STEC (Mughini-Gras et al., 2018b) in the Netherlands, showing that the outcome of classical case-control studies can be greatly enhanced by incorporating source attribution data and *vice versa*. The principle is to first attribute human cases included in a case-control study to sources using the microbial subtyping approach to determine their likely sources, and then to compare the exposures of the attributed cases with those of the controls to identify source-specific risk factors for infection, as well as to infer the underlying transmission pathways.

## Quantitative Exposure/Risk Assessment for Source Attribution

Quantitative Exposure Assessment (QEA) is a bottom-up approach to quantify consumers’ exposure to a pathogen and determine the relative importance of known food sources. Coupling estimates of exposure to the pathogen dose-response relationship, including possible differences in vulnerability of consumer subpopulations, allows for the estimation of associated health risks in terms of predicted number or cases due to the different sources. Potential attribution points with QEA range from primary production to consumption. It is indeed theoretically possible to use this method to assess the relative contributions of different reservoirs, transmission routes, risk factors, etc. In practice, however, QEA is mainly used to assess the relative importance of a few sources to which consumers are directly exposed.

The importance of a given food exposure for pathogen transmission depends on pathogen prevalence and concentration, as well as on food processing and handling conditions and on frequency of consumption. These practices are characterized and their influence on source contamination is assessed (e.g., effects of duration of low-temperature *vs*. ambient storage, consumption of raw/undercooked or thoroughly cooked foods, food handling practices resulting in cross-contamination, etc.). The models developed describe the dynamics of contamination levels along the food production chain and can, therefore, be very complex, accounting for microbial survival, growth, transfer, etc. and requiring a great deal of data from surveys on food consumption, consumer’s habits, etc.

Input data for these models are characterized by natural variability (intrinsic property of the measured data) and uncertainty, reflecting the quality and incompleteness of information (which may be reduced by increasing the number of measurements). These two levels of indeterminacy are usually converted into probability distributions. Data are then introduced in the model using stochastic approaches. Yet, exposure can also be assessed by a simpler deterministic approach multiplying the average consumption of one food per person, the fraction of contaminated portions, the average pathogen concentration in the contaminated portions, and the microbial fraction actually ingested after food preparation. As examples, Evers et al. (2008) estimated the mean number of *Campylobacter* bacteria ingested per person/day to assess the relative importance of 31 routes of transmission from food, direct contact with animals, and the environment in the Netherlands. These authors suggested that raw food consumption and direct contact are the most significant transmission routes of *Campylobacter*. Buettner et al. (2010) have also used a similar approach to estimate the incidence of foodborne campylobacteriosis in Switzerland from contact with pets or travel abroad.

Incorporating probability distributions for the input data in the QEA model often relies on Monte Carlo simulations. When this approach is used to translate the impact of variability on the estimates, the results are expressed as a distribution of exposure or of individual risk (Lake et al., 2011; Opsteegh et al., 2011). The same process can also be performed to quantify the impact of uncertainty expressed as a credibility interval around the exposure or risk estimates (Evers et al., 2008; Buettner et al., 2010). Monte Carlo simulations are sometimes used to simulate, in an undifferentiated way, the impact of both variability and uncertainty (Kosmider et al., 2010). In this case, these two sources of indeterminacy are combined. Otherwise, the impact of variability and uncertainty can be assessed separately using ‘two-dimensional’ Monte Carlo simulations (FDA, 2003). The variability distributions (1st dimension) of individual risk or that of exposure to the hazard are then set in a region of credibility (2nd dimension).

Quantitative Exposure Assessment (QEA) has the advantage of accounting for factors that are difficult to observe with the usual surveillance tools and are therefore complementary with epidemiological methods based on disease surveillance. In theory, QEA allows for source attribution at a very high level of detail/resolution to quantify the contributions of different sources of human infections and the potential impact of targeted interventions. Practically, as this approach requires extensive data on the level of contamination, as well as its evolution along the transmission chain, including data on consumer’s practices and dose-response relationships, the complexity of these models and imperfections in the input data often lead to estimates characterized by high uncertainty.

## Elicitations of Expert Opinion for Source Attribution

In absence or paucity of available data, source attribution can be obtained from experts’ knowledge and opinions, gathered and analyzed through elicitations. Expert elicitation methods described in the literature (Butler et al., 2015b) are generally categorized according to the way experts interact with one another and with the facilitators who collect data at workshops through questionnaires and/or interviews. Each expert is seen as complementary, from the own field of expertise, or traducing a certain kind of variability of individual experience. Keeping experts’ anonymity, without direct expert exchanges, increases independence of expertise, but it does not allow for checking of experts’ correct understanding of questions or answers. Collective workshops may be pressuring for experts, and moderating debates to ensure an equal treatment of diverse, perhaps contradictory opinions, is necessary. The ‘nominal group’ technique is the most commonly used for data collection in structured elicitations of experts at workshops (Delbecq et al., 1975).

Before starting an expert elicitation, the topic of interest has to be defined. A literature review can help identifying where knowledge is lacking and where to target expert elicitations. The questions addressed to experts are prepared by an independent steering group, the members of which do not participate as experts in the elicitation. The steering group takes decision, according to the nature of the questions asked, the elicitation method of choice, and formulate the questions in a language adapted to the experts. The most widely used method for selecting experts is based on the relevance of their competences as shown, for instance, by their record of accomplishments (Efsa, 2014). Experts can also be selected through the non-probabilistic ‘snowball’ sampling method, consisting of randomly drawing possible candidates from the initial target population of experts, then asking to identify colleagues who should be experts in the field. The experts not included in the initial list will complement the group of experts. For the purpose of source attribution, the number of elicited experts varies and has been up to 50 (Butler et al., 2015b).

Participants to the elicitation are informed about the objectives and context of the study and are reminded of definitions of key terms. Training experts on basic concepts of uncertainty estimation is recommended (Efsa, 2014). The level of expertise of the participants in an expert elicitation needs to be assessed. Experts are therefore asked to, e.g., self-assess their expertise by, for instance, indicating where they doubt their ability to provide accurate estimates. The elicitation process can then be serial (e.g., questionnaire distribution following group discussion, or multiple discussions and groups). A ‘series’ of discussions can dispel certain confusions or overcome some points of disagreement between experts. The Delphi method, for example, emphasizes these serial laps of elicitation (Linstone and Turoff, 1975). The opinions of different experts are eventually aggregated with behavioral, mathematical, or mixed methods. In the behavioral aggregation methods, moderated expert discussions end with consensus (i.e., a common estimate). In mathematical aggregation methods, the individual assessments of each expert are aggregated according to value-driven rules, such as measures of central tendency, extremes of data distribution, etc. Mixed approaches allow for interaction between experts and the use of mathematical criteria. Commonly used expert elicitation protocols include:

• The Sheffield protocol, based on a behavioral aggregation of data.
• The Cooke protocol, based on expert ‘calibration’ through exercises, then mathematical aggregation of their numerical assessments.
• The Delphi protocol, based on written answers to a questionnaire, then discussion. A mixed model based on behavioral and mathematical aggregation criteria is then used.

Each stage of expert elicitation is exposed to possible biases. Elicitation by calibrated experts mitigates biases or favors their identification. An overview of published articles on source attribution based on expert elicitation is reported in Table 3.

**TABLE 3:** Overview of source attribution studies by elicitation of expert knowledge.

	**Hoffmann et al., 2007**	**Havelaar et al., 2008**	**Lake et al., 2010**	**Ravel et al., 2010**	**Davidson et al., 2011**	**Vally et al., 2014**	**Butler et al., 2015a**	**Hald et al., 2016**
Expert selection method	Snowball	Unspecified	Publications	Snowball	Snowball	Publications	Snowball	Snowball
Number of experts enrolled	42	16	14	54	135	12	31	72
Data collection method	Mail	E-mail	Workshop	Mail	Mail	Workshop	E-Mail	E-Mail
Assessment of level of expertise	Self-assessed	Self-assessed	Unspecified	Self-assessed	Self-assessed	Unspecified	Self-assessed	Unspecified
Serial elicitation	No	No	No	No	No	Yes	Yes	No

## Natural Experiments

Several ‘epidemiological disasters’ involving poultry and *Campylobacter* have served as natural experiments of the effect of a major and sudden reduction of consumer’s exposure to *Campylobacter*. For instance, in 1999, the dioxin crisis in animal feed in Belgium led to the national withdrawal from the market of various poultry products. Concurrently with this withdrawal, there was a drastic reduction in the nationwide consumption of chicken meat, which was associated with a drop of 40% in campylobacteriosis in Belgium (Vellinga and Van Loock, 2002). Similarly, in 2003, an epidemic of avian influenza (H7N7) hit the Netherlands. To control this epidemic, massive bird culling measures targeting predominantly laying hens were implemented, and several poultry slaughterhouses were closed. This was associated with concurrent declines in campylobacteriosis, locally and nationally, by 30–50%, depending on the province (Friesema et al., 2012). Although sales of poultry meat declined, this alone could not explain the reduction in campylobacteriosis, which continued far beyond the recovery in poultry meat sales. Overall, this natural experiment has suggested that a significant fraction of campylobacteriosis cases could be prevented by reducing the environmental burden of *Campylobacter* originating from poultry. Unfortunately, such natural experiments only allow for the retrospective observation of effects. The implementation of national intervention programs to reduce *Campylobacter* on poultry meat, however, allows for prospective opportunities to study the effect of reduced exposure to poultry-associated *Campylobacter*. For instance, following interventions in New Zealand, the total number of campylobacteriosis cases decreased by 54% (Sears et al., 2011).

## Choosing the Appropriate Source Attribution Method

**FIGURE 5:**
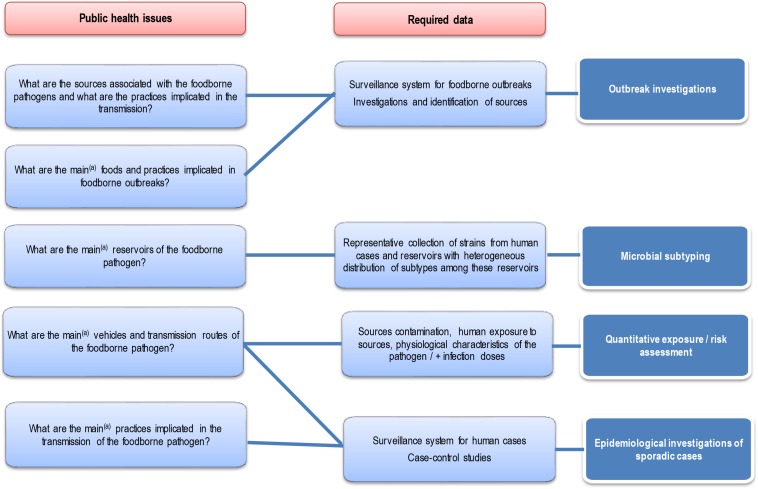
Preferential choice of source attribution methods based on public health issues. ^(a)^Ranking and/or quantifying the relative importance.

Table 4 and Figure 5 provide an overview of the elements guiding the choice of a source attribution method. The choice of the source attribution method depends on the point of attribution across the farm-to-fork continuum, the quality/completeness of data available and the characteristics of the pathogen in question, including data on subtyping and microbial fitness (e.g., plasticity, clonality, diversity, pathogenicity, etc.). The specific public health issues to address may also guide the choice of the methods (Figure 5). For instance, epidemiological methods like case-control studies are best suited to attribute sporadic cases ‘downstream’ to specific food exposures, including transmission routes and risk factors. On the other hand, microbiological methods, such as the frequency-matching models, can also attribute sporadic cases up to the level of reservoir, requiring data on pathogen subtyping (for humans and sources), as well as data on prevalence and exposure (for sources), with additional data on pathogen fitness being a potentially useful piece of information to incorporate in the models. As exposure sources are dynamic in nature and may change rapidly, timeliness is an important feature of source attribution studies. Methods that are more suited to detect changes over time (e.g., those based on the microbial subtyping approach) are therefore also more suited to evaluate the impact of control strategies, as exemplified in the aforementioned *Campylobacter*-control intervention in New Zealand (Sears et al., 2011). Yet, most of the methods described here depend on the stability of source data to be reliably interpreted. While methods to minimize the consequences of having to use non-recent (and non-local) data have been proposed (Smid et al., 2013), timeliness should be more directly called out as a design feature of source attribution studies. As an example concerning the analysis of outbreak data, in the United States of America, the Interagency Food Safety Analytics Collaboration (IFSAC) has incorporated a process to weight more recent outbreak data to reflect changes in underlying patterns of risk.

**TABLE 4:** Overview of the main characteristics and necessary data for source attribution methods.

	**Point of attribution**				**Pathogen data**		**Human data**			**Source data**		
				
**Method**	**Reservoir^∗^**	**Exposure**	**Transmission route**	**Risk factor**	**Subtyping**	**Fitness**	**Subtyping**	**Sporadic or epidemic status**	**Exposure & risk factors^∗∗^**	**Subtyping**	**Prevalence and exposure^∗∗∗^**	**Need to consider all potential sources**
Epidemiological (case-control study)	No	Yes	Yes	Yes	No	No	No	Sporadic	Yes	No	No	High
Epidemiological (outbreak investigation)	No	Yes	Yes	Yes	No	No	No‡	Epidemic	Yes	No	No	High
Microbiological (frequency-matching models)	Yes	Yes	Yes	No	Yes	No‡	Yes	Sporadic and epidemic	No‡	Yes	Yes	Medium
Microbiological (population genetics models)	Yes	Yes	Yes	No	Yes	No	Yes	Sporadic or epidemic	No	Yes	No	High
Quantitative exposure/risk assessment	Yes	Yes	Yes	Yes	No	Yes	No	N/A	Yes	No	Yes	Medium
Expert elicitations	Yes	Yes	Yes	Yes	No	No	No	Sporadic or Epidemic	No	No	No	Low

*N/A, not applicable. ^∗^Or amplifying hosts. ^∗∗^Including travel status. ^∗∗∗^In this case, food consumption weights. ^‡^But can be used if available.*

For microbiological methods, the sampling point of the sources essentially determines the point of attribution (Mughini-Gras et al., 2014a). The use of these methods is not suited to pathogens with low genotypic or phenotypic diversity (i.e., non-heterogeneous distribution) among the source. Furthermore, for frequency-matching models, the subtypes upon which the attribution relies must possess some stability along the farm-to-fork continuum, as they are often compared between primary production and human cases. If this is not the case, population genetics models, which account for evolutionary processes in the genetic targets investigated, are to be preferred. In general, microbiological methods for source attribution are data-intensive and their use is often conditional to the existence of well-established integrated surveillance systems, as well as the systematic and harmonized application of subtyping methods that are sufficiently discriminating for human and source strains, but also harmonized metadata linked to these strains. These methods are particularly helpful in quantifying the relative contributions of several putative sources of human sporadic infections, with the relevance of the results being also dependent on the number of sources, including the distinction between imported and domestic foods, and the information taken into account in the models. Moreover, if surveillance data are available for multiple time periods, attributions can be performed over time, making it possible to monitor trends in the contributions of different sources and to evaluate the impact of interventions that target one or part of the sources. Population genetics models require a representative collection of strains from human cases, and sources and no information on exposure is in principle needed. Yet, to limit erroneous attributions, the panel of potential sources included in the analysis is critical and needs to be as complete as possible, as no fraction of non-attributable cases is contemplated, with each case being assigned a probability to originate from each source based on the genetic proximity to the source strains.

Quantitative Exposure Assessment models can estimate the proportion of cases attributable to sources at all levels of the food production chain. These models, however, require extensive data on source contamination, exposure and microbial characteristics. The availability of relevant (e.g., up-to-date, local, pathogen-specific, etc.) input data is the main limitation of QEA. Moreover, estimating the number of cases attributable to various sources based on pathogen prevalence, concentration and exposure (food consumption) often means ignoring which strains of this pathogen are actually relevant to public health, i.e., which are those that infect (and are therefore pathogenic for) humans. Indeed, not all strains of a pathogen that contaminate a source are responsible for the human disease burden. Differences among strains in their virulence and survivability in certain sources are difficult to be accounted for in QEA models, as the latter are generally built to estimate how much pathogen (as a whole) reaches the human population, but not the fraction that actually causes disease, as addressed in a recent paper (Fritsch et al., 2018). Moreover, QEA models do not account for host-related conditions (e.g., comorbidities, impaired immune system, etc.) predisposing to disease. These conditions are intrinsically accounted for when starting from what we see in humans and trying to trace back their sources by looking at, e.g., the associated risk factors or the distribution of strains in the sources. Finally, expert elicitations can be used to attribute all types of cases to any point and do not require any data. Indeed, they should be performed when no data are available. The main limitation of this method is that it relies only on opinions and depends on the quality of the expert panel recruited. While data availability and quality are the main factors that guide the selection of the applied methods, the specific public health issues to address may also guide the choice of the methods (Figure 5).

In conclusion, a variety of source attribution approaches have been applied in recent years. Each approach has its own advantages and disadvantages, including some unaddressed methodological challenges, to be considered in a systematic way when performing and interpreting a source attribution analysis, or when developing a new source attribution model. New insights from WGS, such as identifying previously unknown (or under/overrated) sources for a specific pathogen will add new levels of complexity. As there is clearly no single approach that satisfies all needs, different methods may be combined or at least applied in a comparative way.

## Author Contributions

This review is part of an expert work carried out by the ANSES working group on Source Attribution of Foodborne Diseases (https://www.anses.fr/fr/content/avis-et-rapport-de-lanses-relatif-%C3%A0-lattribution-des-sources-des-maladies-infectieuses). This working group was coordinated by PK. LW and MS supervised the project. LM-G wrote the manuscript draft with input from all other authors.

## Conflict of Interest

The authors declare that the research was conducted in the absence of any commercial or financial relationships that could be construed as a potential conflict of interest.

## Footnotes

1 https://www.pasteur.fr/sites/default/files/veng_0.pdf

2 https://www.cdc.gov/pulsenet/pathogens/pfge.html
